# Exploring the concept of patient centred communication for the pharmacy practice

**DOI:** 10.1007/s11096-017-0508-5

**Published:** 2017-09-09

**Authors:** Majanne Wolters, Rolf van Hulten, Lyda Blom, Marcel L. Bouvy

**Affiliations:** Division of Pharmacoepidemiology and Pharmacotherapy, Faculty of Science, Utrecht Institute for Pharmaceutical Sciences, PO Box 80082, 3508 TB Utrecht, The Netherlands

**Keywords:** Communication, Patient centredness, Pharmaceutical care, Pharmacy practice, UMPA model

## Abstract

*Background* Patient centred communication can improve pharmaceutical care, but is not well described for pharmacists. *Aim of the review* To provide a comprehensive and accessible overview of the concept of patient centred communication for the pharmacy practice. *Method* A scoping review and thematic analysis was undertaken to synthesize the extracted data and present it in a model. *Results* Literature search and selection resulted in eighteen articles. Thematic analysis of the extracted data led to five categories regarding patient centred communication. Two categories refer to phases of a pharmaceutical consultation: (1) shared problem defining and (2) shared decision making; three refer to underlying concepts and assumptions about patient centredness regarding (3) the patient, (4) the pharmacist and (5) the therapeutic relation. The categories were modelled in the so called Utrecht’s Model for Patient centred communication in the Pharmacy. *Conclusion* Although there might be barriers to implement patient centred communication in the pharmacy, the concept of patient centred communication as described in the literature is relevant for the pharmacy practice.

## Impacts of practice

- Training of pharmacy staff in patient centred communication may be helpful in addressing drug related problems and achieving better health outcomes.
- Patient centred communication by pharmacists may enhance their role of care giver.

## Introduction

Communicating with patients about their experiences, needs and concerns regarding their health and medication is essential to identify drug related problems such as overuse, adverse drug reactions and non-adherence [1]. Such communication is an important part of pharmaceutical care: care which urges pharmacists to take responsibility for the clinical outcomes of drug therapy by preventing, identifying and resolving drug related problems [2].

This was also explicitly recommended in a recent Cochrane review. This review suggests that solely providing information or education appears ineffective to improve adherence or clinical outcomes [3]. There is evidence that successful interventions combined patient education with counselling [4]. In pharmacy practice different kinds of interventions can be distinguished, such as counselling when dispensing drugs or medication use review services. Studies have shown that cognitive pharmaceutical services improve the quality of drug therapy and outcomes of several chronic diseases [5–8]. However, the improved quality of drug therapy does not always seem to lead to better patient outcomes. This may be caused by factors concerning the illness or the patient’s lifestyle, but also by difficulties that pharmacists experience with exploring patients’ needs and concerns [9–11]. Almost half of the studies about patient-provider interactions in pharmacy practice focussed solely on information giving [12]. All this suggests that patient communication in pharmacy practice needs improvement. The concept of patient centred communication is widely advocated as a way to improve communication [2, 13, 14].

## Aim of the study

Patient centred communication is extensively described and studied regarding doctors and nurses, but it is not well defined for pharmacists. Bensing states that patient-centredness is an ambiguous and multidimensional concept, which is interpreted differently by individual caregivers [15]. Therefore it might be difficult for pharmacists to properly understand patient centred communication. The aim of this study is to provide a comprehensive and accessible overview of the concept of patient centred communication for the pharmacy practice.

## Methods

A scoping review was conducted. Scoping reviews ‘aim to map *rapidly* the key concepts underpinning a research area and the main sources and types of evidence available’ [16] As Arksey and O’Malley stated, a scoping review is suitable to summarize and disseminate research findings to professionals, which is in line with the aim of our research (Table 1) [17, 18]. Table 1 contains a detailed description of the six different phases of our scoping review, starting with phase 1: determining the aim of the review.

**Table 1:** Phases of the scoping review as performed in this study [1, 2]

Phases	Detailed description
1. Identifying the aim and research question	Aim: provide a comprehensive and accessible overview of the concept of patient centred communication for the pharmacy practice
2. Identifying relevant studies while considering the balance between feasibility and comprehensiveness	Search strategy Cochrane search: MESH descriptor ‘patient centered care’ (12 Feb 2012) Pubmed search: MESH major topics ‘patient centered care’ AND ‘communication’ (21 Feb 2012) Pubmed search: key words ‘patient cent(e)red care’ AND ‘communication’ (21 Feb 2012)
3. Study selection by a team of reviewers	Selection of eligible articles a. Screening on title and abstract according to the inclusion and exclusion criteria (MW and LB) b. Screening full text determining conceptualization of patient centred communication or describing a measurement instrument for patient centred communication which refers to an underlying conceptualization (MW, LB, RvH) c. Snowballing on the selected articles (MW)
4. Charting the data	a. Extraction of descriptions of patient centred communication from the selected articles
5. Collating, summarising and reporting the results with implications for practice, policy or research	a. Thematic analysis by open coding (by MW and RvH by hand); (iterative process) b. Description of the different themes c. Discussion of the relations between the different themes and defining the main categories (by MW and RvH) d. Presentation of the different categories and underlying themes in a concept -model
6. Consultation of stakeholders on the results	a. Presentation and discussion of the concept model individually with 6 community pharmacists, who work as teachers or researchers at Utrecht University b. Suggestions were processed in the final model

In phase 2 a literature search was performed in Cochrane and Pubmed database (respectively 12 and 21 February 2012) using a limited amount of search terms in order to narrow the amount of references but still being able to identify the main authors on patient centred communication.

**Fig. 1:**
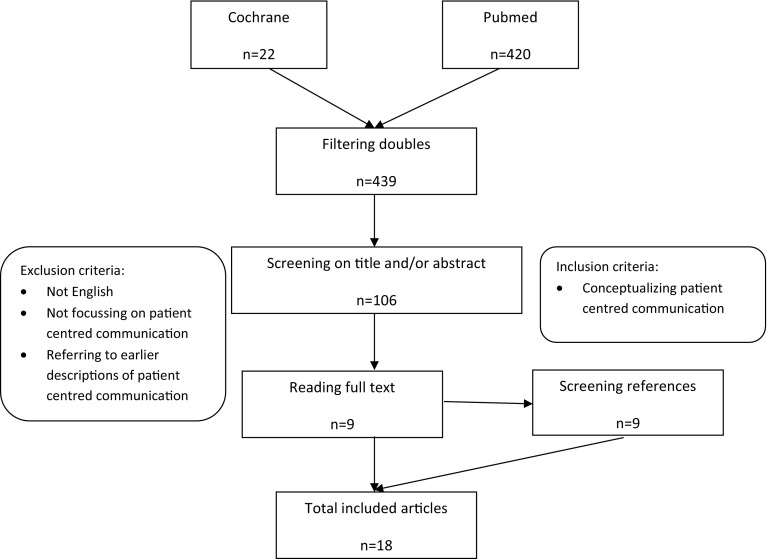
Flowchart of literature search and selection

For the selection of relevant articles (phase 3), articles were screened judging whether they focused on communication between health care provider and patient in general and were published in English (based on title and abstract). Excluded were articles about inter-professional communication, e-health, management, aspects of patient centred care other than communication or on specific groups (patients, diseases, gender, culture) (see Fig. 1 flow chart).

Subsequently, full-text articles were read to determine whether they conceptualized patient centred communication (inclusion criterion). Articles were excluded when referring to older articles and not giving a different or more elaborate view on patient centred communication than the earlier article.

Finally, through snowballing to earlier publications by examining the full-text articles for relevant references, articles were included that gave a different or additional view on patient centred communication.

In phase 4 the text segments of the descriptions of patient centred communication were extracted from the selected articles. In phase 5 a thematic analysis of the data was done until agreement was reached on all the themes/categories. The different categories were interconnected to one another and presented in a model. Finally, in phase 6 stakeholders were interviewed individually for feedback on this model.

## Results

### Literature search and selection

The literature search and selection resulted in eighteen articles describing the concept of patient centred communication (Fig. 1).

### Thematic analysis including interrelating of the themes

From these articles the descriptions of patient centred communication were extracted (See “Appendix 1”.)

The thematic analysis of the data led to a total of 21 themes (Table 2). Combining similar themes led to five main categories. Category 1 and 2 refer to two sequential phases of the consultation process: the shared problem defining (cat.1) and the shared decision making (cat. 2). The other categories describe concepts or assumptions about patient centredness and are related to the patient (cat. 3), the health care provider (cat. 4) or the therapeutic relationship (cat. 5).

**Table 2:** Overview of the categories describing patient centered communication and their interconnectedness

The patient centered consultation	
*1 Shared problem defining* [19–23]	
1.1 Involve the patient in the consultation [20, 21, 23–28]	
1.2 Explore and understand the patient’s perspective [19–22, 24–26, 28–32]	
1.3 Consider patient’s situation [24, 27, 28, 33, 34]	
*2. Shared decision making* [20, 22–24, 26, 27, 30, 32, 33, 35, 36]	
2.1 Inform the patient [20, 21, 25–28, 31, 33, 34]	
2.2 Consider options and preferences [23, 25, 27, 28, 31, 33]	
2.3 Choose management plan [19, 21, 22, 28, 31, 33]	
2.3.1 Action planning [21, 24, 27–29]	
2.3.2 Enable self-management [19, 23, 30, 31, 35]	
2.3.3 Agreement check [21, 24, 29]	

Underlying concepts and assumptions about patient centredness	
*Related to the patient*	
3.1 Biopsychosocial perspective [21, 22, 35, 36]	
3.2 Patient as a person [20, 32, 35, 36]	
3.3 Health promotion [19, 20, 32]	
*Related to the health care provider*	
4.1 The health care provider as a person [21, 24, 29, 36]	
4.2 Required skills [23, 27, 34, 35]	
4.3 Empathy (open to emotions) [23, 25–28, 31]	
*Related to the therapeutic relation*	
5.1 Building a relation [19–21, 27, 29, 32, 34]	
5.2 Therapeutic alliance [31, 35, 36]	
5.3 Trust [27, 31, 34]	
5.4 Handling within the given context [19, 32]	

In the next paragraphs these five categories (and underlying themes) are described in more detail.

### The patient centred consultation

#### Category 1: Shared problem defining

The concept of ‘shared problem defining’ describes the process of exploring and understanding the patient’s view. The outcome of this process is a shared understanding and agreement of the pharmacist^1^ and patient on the problem(s) that need to be dealt with during the consultation [19–23].

The problem-defining process includes the following steps. At first, at the start of the consultation the pharmacist (by active listening including questioning) encourages the patient to be involved and thereby enhances the relationship with the patient [20, 21, 24–28]. This stimulates patients to express their expectations of the visit, their problem(s) and concerns [23, 25, 28].

Next the pharmacist further explores the patient’s perspective using active listening and summarizing [21, 24, 26, 29]. Different topics may be addressed: patient’s needs and concerns, practical problems, patient’s knowledge and expectations about health and treatment or the influence of the illness and/or therapy on their life [19–22, 24–26, 28–32].

Subsequently the pharmacist considers the patient’s situation and shares their expert opinion [24, 28, 33, 34]. To be sure that the patient comprehends the pharmacist’s perspective on the patient’s problems, the pharmacist has to give time for the patient to process the information and to ask questions to be able to respond to the pharmacist’s perspective [27, 28]. Shared problem defining may enhance the role of pharmacists: their expert knowledge may add to the patient’s perspective. In addition the pharmacist may identify possible drug related problems the patient is not aware of.

#### Category 2: Shared decision making

The concept of shared decision making developed alongside the concept of patient centred communication and is extensively described as such. Several authors view shared decision making as an element of patient centred communication [20, 23, 24, 32, 33, 35]. Shared decision making is an approach whereby the pharmacist encourages the patient to actively participate and thus shares power and responsibility [22, 30, 36, 37]. However, the pharmacist has to consider the extent to which patients want to be involved in choices [20, 22, 26, 27].

Patients need to be well informed, both before and during treatment in order to be able to make an informed decision about treatment of their illness [20, 21, 25–28, 31, 33, 34]. The pharmacist should check for comprehension and patients’ information needs, give clear explanations and encourage questions [20, 21, 27, 28, 31, 33].

Often there are different ways to treat and manage a disease. Both the therapeutic options and patients preferences should be taken into account by both parties [25, 27, 33]. The patient may have specific requests, experience (practical) barriers or is ambivalent [23, 25, 28]. The pharmacist, being an expert, can give information and advice about the different (treatment) options, keeping in mind the patient’s level of self-efficacy [23, 27, 31].

Finally, the patient and pharmacist should reach an agreement on a management plan, which is concordant with the values of the patient [19, 21, 22, 28, 31, 33]. Reaching an agreement includes the following three aspects.

Firstly, the pharmacist and patient should consider the feasibility of the chosen solution. They have to discuss the practicality of the plan, the follow up and plan for the unexpected [21, 24, 27–29]. Secondly, the pharmacist enables and encourages the patient to take responsibility for the self-management of the disease [19, 23, 30, 31, 35]. Lastly, the pharmacist can summarize the agreements and ask for feedback, in order to check agreement [21, 24, 29].

### Underlying concepts and assumptions

The following concepts and assumptions about patient centredness underpin and support the patient centred consultation and refer to the patient, the pharmacist and the relationship.

#### Category 3: Related to the patient

The pharmacist should consider patients from a biopsychosocial perspective, and not solely from a biomedical perspective. [21, 22, 35, 36]. Secondly, pharmacists adapt their care taking into account how the illness and medication affect the individual patient. This is described in terms as understanding the whole person, the patient as a person, or a holistic approach [20, 32, 35, 36] Thirdly, the idea of health promotion fits in with this: not only treating the presented disease, but considering the health and quality of life of the patient in total, now and in the future [19, 20, 32].

#### Category 4: Related to the pharmacist

The concept pharmacist-as a-person relates to two aspects. Firstly, that it matters to patients who the pharmacist is as a person [21, 36]. Secondly, pharmacists need to take care of themselves and reflect on their own feelings, values and actions (reflective practice) [21, 24, 29]. The pharmacist needs to be competent, not only in pharmacotherapy, but also in communication skills [23, 27, 34]. Special attention is given to being empathetic, because this is essential for building an effective relationship [23, 25–28, 31].

#### Category 5: Related to the therapeutic relationship

A pharmacist should be able to establish a relationship with the patient to have an effective consultation [19–21, 27, 29, 32, 34]. This therapeutic relationship is not only a prerequisite to a patient centred consultation. The relationship in itself can have a therapeutic effect [31, 36]. Horvath et al. [38] described the therapeutic alliance as the emotional bond developed between a health professional and patient that allows the patient to make therapeutic progress. Trusting the pharmacist is an important aspect of the therapeutic relationship. Patient’s trust in the pharmacist and confidence in their expertise will help to trust the proposed treatment [27, 31, 34].

Although it is important to take the wishes of the patient into account, the pharmacist and patient have to act within the given context. This means being realistic about time and resources and taking into account (personal) limitations and requirements [19, 32].

### The UMPA model

**Fig. 2:**
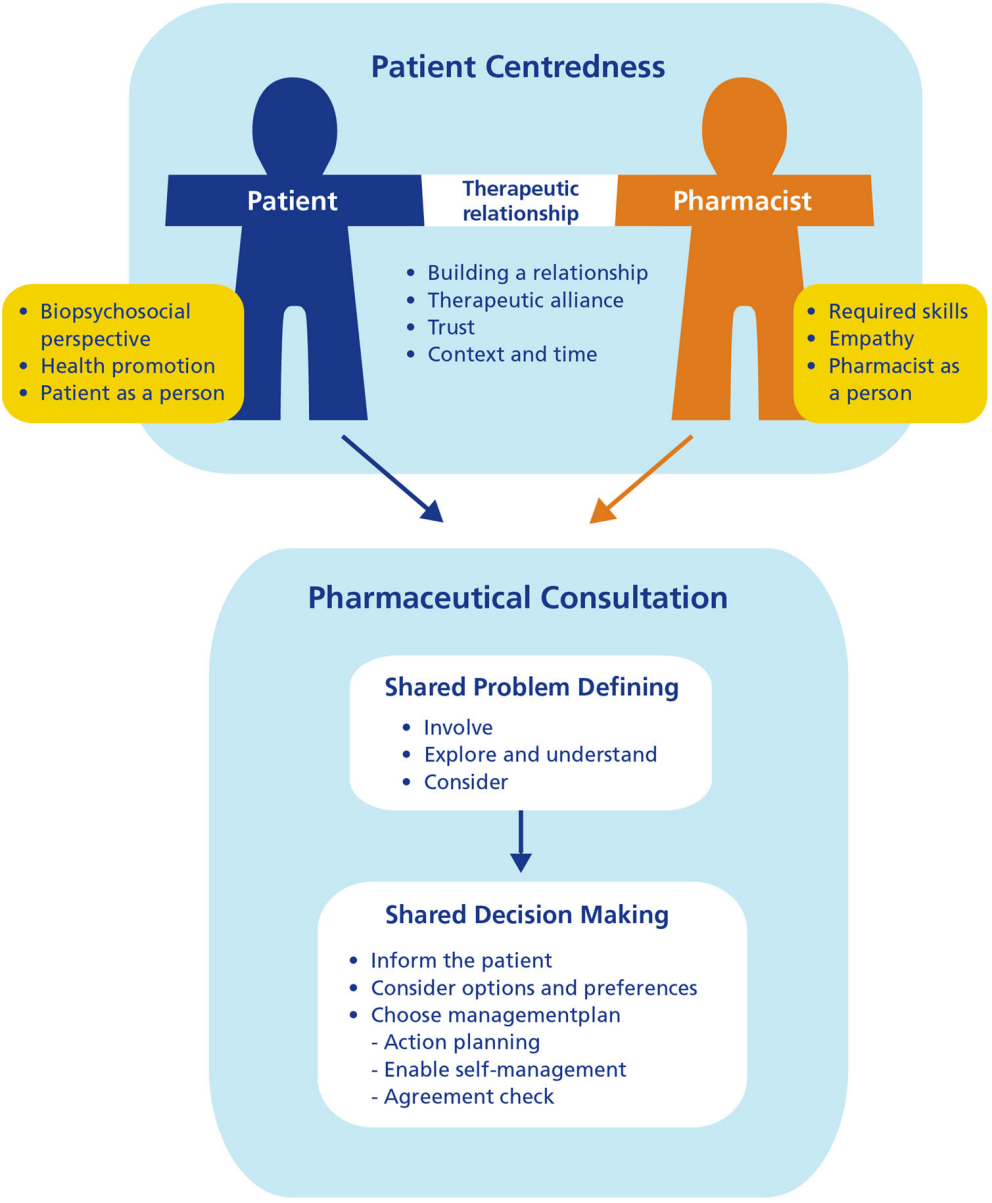
UMPA: Patient centred communication in the pharmacy about drug related problems

The categories and underlying themes were modelled in the so called Utrecht’s Model for Patient centred communication in the Pharmacy 2 to give an accessible overview (Fig. 2. UMPA model). The consultation with stakeholders led to minor changes in design and wording.

## Discussion

The current study provides an overview of patient centred communication, based on the descriptions found in eighteen publications. The different descriptions of patient centred communication have been translated to pharmacy practise and are presented in a model for the pharmaceutical consultation, the so called UMPA-model (Utrecht’s Model for Patient centred Communication in the Pharmacy). Central in this model are shared problem defining and shared decision making. These are supported by assumptions about patient centredness regarding the patient, the pharmacist and the therapeutic relationship.

The six phases of a scoping review were performed as described by Arksey and O’Malley and by Levac [17, 18]. Although a limited literature search was done, we argue that the main descriptions on patient centred communication were identified, because of extensive snowballing until saturation.

The process of study selection and the thematic analysis was done by a small team, therefore there might be a risk of bias. The feedback of the stakeholders however showed that the model has face value.

When describing patient centred communication there is the risk of oversimplifying and consequently not doing the concept justice [39]. However, the included data were rich: some authors focussed on the consultation and practical skills (e.g. Makoul et al.) [21], others more on theoretical notions on patient centred communication (e.g. Mead and Bower) [36], sometimes it was mixed (e.g. Epstein and Street) [31]. We did justice to the complexity by using all data.

The included articles described patient centred communication mostly for doctors or for health care providers in general. We argue that it is also applicable to pharmacy practice.

Firstly, pharmaceutical patient care aims at optimising the outcomes of drug treatment for the individual patient. When a patient experiences problems with medication, a pharmacist can help to solve these problems by communicating with the patient (e.g. by giving information, reassurance or advice, by motivating or by solving (practical) problems).

Secondly, although one may question to what extent pharmacists are involved in shared decision making, which often seems to refer to decisions on treatment [22, 25, 27], in our view pharmacists have a specific role as health provider and they as well as other health professionals help patients to make decisions regarding treatment, e.g. managing the drug regimen. We argue that a pharmacist can play an active role in defining the specific drug problems and solve them together with the patient, which is in line with the two phases of a patient centred consultation.

Finally, in the pharmacy practice pharmacists monitor and evaluate patient’s medication use and therefore they can signal possible problems of which the patient is not aware. Therefore it might be that the consultation does not start with a request for help from the patient, but with the pharmacist exploring whether there is a problem, e.g. under consumption. However, none of the authors stated that it is a requisite for patient centred communication that the patient always have to bring in the problem theirself. And even so, especially in this kind of situations the pharmacist has to take the steps of shared problem defining to determine together with the patient what the exact problem is, which makes the need for patient centred communication even bigger.

In our view these characteristics that are specific for pharmaceutical patient care, do not change the suitability of patient centred communication to pharmaceutical patient care.

Nevertheless one could argue that there are practical objections that might hinder patient centred communication in the pharmacy practice. Firstly, it could be more difficult to build a relationship with patients because they communicate with different staff members. Secondly, patients may be reluctant to discuss their problems, due to the poor privacy conditions in pharmacies. Thirdly, communication may be hampered when staff is busy and do not seem to have time for consultation. Fourthly, patients do not always collect the medication themselves which may limit the communication about patients’ drug problems/questions. Lastly, patients may be unaware of the possible support of the pharmacy staff to resolve their drug related problems. Therefore they do not always have an explicit wish for help or consultation of the pharmacist.

Pharmacists have to put more effort into connecting with the patient to overcome these barriers to patient centred communication. Therefore training of pharmacy staff is useful, but it is also important to rethink the organisational process of preparing and delivering medications to patients in the pharmacy. All this does not proof the concept of patient centred communication less valuable for the pharmacy practice, but implementing patient centred communication in practice needs attention to overcome these obstacles.

## Conclusion

Patient centred communication is a new concept for the pharmaceutical consultation. According to the literature, it refers to both the consultation process with the phases of shared problem defining and shared decision making, and to underlying concepts and assumptions regarding the patient, the pharmacist and their relationship. All themes from the thematic analysis seem to be relevant for the pharmaceutical practice, although there might be barriers to implement patient centred communication in the pharmacy. The UMPA-model can be helpful in presenting patient centred communication and supporting (future) pharmacists to understand the requirements for patient centred pharmaceutical care, not only as a practical set of communication skills or phases in a consultation, but also as a principle and attitude towards pharmaceutical care.

## Appendix 1

See Table 3.

**Table 3:** Thematic analysis of the dataset

Themes (1–21)	References	Text segment no.^a^
*1/Shared problem defining*		
Achieve a shared understanding [of the problems with the patient]	[19]	9
Partnership [finding common ground [..] and mutual agreement about patients’ ideas, the problem]	[20]	51a
Reach agreement on problems [..]	[21]	48a
Reaching a shared understanding of the problem [..] with the patient that is concordant within the patient’s values	[22]	73a
Patient’s involvement in the problem-defining process	[23]	56a
*2/Involve the patient in the consultation*		
Patient’s involvement in the problem-defining process [encouraging full expression of problem(s) and expectations of the visit]	[23]	56b
Communication [listening, requirements for information]	[20]	50b
Allow patients to express their major concerns	[25]	19
Relationship: let the patient talk	[24]	30
Gather information [actively listening using nonverbal and verbal techniques]	[21]	45
Explores the patient’s view by actively listening, and clarifies the reasons for help	[27]	88
Encourages the patient to respond to the questions asked[..]	[27]	89a
[..] facilitation of patient disclosure	[26]	67a
Partnership building [through active enlistment of patient input]	[26]	69a
Invest in the beginning [show familiarity, question style, expansion of concerns, elicit full agenda]	[28]	75
*3/Explore and understand the patient’s perspective*		
Define the reason for attendance, including the history, the patient’s ideas, concerns and expectations, and the effects of the problem	[19]	6
Exploring both the disease and the illness experience	[32]	13
Elicit patients' explanations of their illnesses	[25]	21
Ability to elicit and discuss patients’ beliefs	[30]	54
Prior to the consultation: how has the patient prepared for the visit? [what does he/she expect]	[24]	29
Anxieties: what does the patient want?	[24]	31
Open the discussion [elicit the patient’s full set of concerns]	[21]	44b
Communication [exploration of concerns]	[20]	50c
Elicit the Patient’s perspective [patient’s understanding of problem, goals for visit, impact on life]	[28]	76
Eliciting and understanding the patient’s perspective: concerns, ideas, needs, feelings and functioning	[22]	71
Data gathering [..]	[26]	67b
Summarizing; obtaining a sufficiently comprehensive idea of the patient’s real reason for consulting you.	[29]	7
Common language: GP’s summary.	[24]	32
Understand the patient’s perspective	[21]	46
Exchanging information [understanding what patients know and believe about health]	[31]	80a
*4/Consider patient’s situation*		
Both the health care provider^b^ and patient share information with each other	[33]	26a
Translating: from lifeworld to world of medicine	[24]	33
Knowledge and professionalism	[34]	95
Invest in the end [give clear explanations, test for comprehension, encourage questions, use patient’s frame of reference, allow time to absorbe]	[28]	78a
Encourages the patient to respond to [..] the information given, and the diagnosis	[27]	89b
*5/Shared decision making*		
Shared decision making	[35]	63
Finding common ground regarding management	[32]	15
Both the health care provider and patient are involved (in the treatment decision-making process)	[33]	25
Interaction: negotiation on what to do	[24]	34
Sharing power and responsibility	[36]	40
[..] to active the patient to take control in the consultation [..]	[30]	55a
Patient’s involvement in the decision-making process	[23]	57
Patient involvement	[35]	61
Helping patients to share power and responsibility by involving them in choices to the degree that they wish	[22]	74
Partnership [finding common ground – exploration, discussion and mutual agreement about treatment]	[20]	51b
Partnership building [through active enlistment of patient input]	[26]	69b
Encourages patients to actively participate in decision-making	[27]	87
*6/Inform the patient*		
Both the health care provider and patient share information with each other	[33]	26b
Give patients information	[25]	23
Share information [use language the patient can understand, check for understanding, encourage questions]	[21]	47
Patient education [..]	[26]	68b
Exchanging information [patients’ information needs, communicating clinical information]	[31]	80b
Delivers and organizes information, and systematically checks that the information is well understood	[27]	91
Transparency of progress and outcome	[34]	97
Communication [clear explanation]	[20]	50d
Invest in the end [give clear explanations, test for comprehension, encourage questions]	[28]	78b
*7/Consider options and preferences*		
Both health care provider and patient take steps to participate in the decision-making process by expressing preferences	[33]	27
Advises the patient about possible treatment options and helps the patient to make choices	[27]	90
Involve patients in developing a treatment plan	[25]	24
Seek patients' specific requests	[25]	20
Exchanging information [(sharing bad news) and prognostic information]	[31]	80c
Consideration of the patient’s ambivalence or self-efficacy	[23]	59a
Invest in the end [explore barriers]	[28]	78c
*8/Choose management plan*		
A treatment decision is made and both the health care provider and patient agree on the treatment to implement	[33]	28
Reach agreement on [..] plans	[21]	48b
Making decisions	[31]	81
Reaching a shared understanding of the [..] treatment with the patient that is concordant within the patient’s values	[22]	73b
Choose an appropriate action. [with the patient for each problem]	[19]	8
Invest in the end [involve in decisions]	[28]	78d
*9/Action planning*		
Converting insight into action: from consultation to everyday life	[24]	35
Discusses the practicality of the therapeutic plan	[27]	92
Invest in the end [explore plan acceptability]	[28]	78e
Provide closure [..], discuss follow up]	[21]	49a
Invest in the end [plan for follow-up]	[28]	78f
Safety-netting; planning for the unexpected	[29]	4
*10/Enable self-management*		
Enablement	[35]	66
Enabling patient self-management	[31]	83
Involve the patient in management. [and encourage him/her to accept appropriate responsibility]	[19]	10
Ability to active the patient to take control [..] in the management of their illness	[30]	55b
Consideration of the patient’s [..] self-efficacy	[23]	59b
*11/Agreement check*		
Agreement check: safety netting	[24]	36
Handing-over; making sure the patient is happy with the outcome of the consultation	[29]	3
Provide closure [summarize and affirm agreement with the plan of action, [..]	[21]	49b
*12/Biopsychosocial perspective*		
Biopsychosocial perspective	[36]	38
Building a relationship [approach to care, which emphasizes both the patient’s disease and his or her illness experience]	[21]	43a
[..] Biopsychosocial perspective	[35]	64a
Understanding the patient within his or her unique psychosocial context	[22]	72
*13/Patient as a person*		
Understanding the whole person	[32]	14
Understanding the whole person	[20]	53
Holism/[..]	[35]	64b
Patient-as-person	[36]	39
*14/Health promotion*		
Incorporating prevention and health promotion	[32]	16
Health promotion	[20]	52
Consider other problems, including continuing problems and risk factors	[19]	7
*15/The health care provider-as-person*		
Health care provider-as-person	[36]	42
Housekeeping; taking care of yourself	[29]	5
Leave from consultation: time for reflection	[24]	37
Building a relationship [requires an awareness that ideas, feelings, and values of [..] the health care provider influence the relationship]	[21]	43b
*16/Required skills*		
Health care provider’s picking up the patient’s cues	[23]	58
Skills	[35]	62
Uses communication skills effectively	[27]	86
The ability to communicate	[34]	93
*17/Empathy (open to emotions)*		
Facilitate patients' expressions of feeling	[25]	22
[..] counseling	[26]	68a
Emotionally responsive communication	[26]	70
Health care provider’s overall responsiveness to the patient	[23]	60
Demonstrate empathy [encourage emotional expression, accept feelings, identify feelings, show good nonverbal behavior]	[28]	77
Responding to emotions	[31]	79
Creates effective therapeutic relationships with patients [shows concern with patients (and families)]	[27]	85a
*18/Building a relation*		
Connecting; achieving a working rapport with the patient; getting on the same wavelength	[29]	1
Open the discussion [establish/maintain a personal connection]	[21]	44a
Establish or maintain a relationship	[19]	12
Communication [health care provider –patient relation]	[20]	50a
Enhancing the health care provider-patient relationship	[32]	17
Building a relationship [requires an awareness that ideas, feelings, and values of both the patient and the health care provider influence the relationship]	[21]	43c
An understanding of people and an ability to relate	[34]	96
Creates effective therapeutic relationships with patients	[27]	85b
*19/Therapeutic alliance*		
Fostering healing relationships	[31]	82
Therapeutic alliance	[36]	41
Relation—knowing the health care provider	[35]	65
*20/Trust*		
Confidence	[34]	94
Managing uncertainty	[31]	84
Creates effective therapeutic relationships with patients [creates trust]	[27]	85c
*21/Handling within the given context*		
“Being realistic” about personal limitations and issues such as the availability of time and resources	[32]	18
Use time and resources appropriately, during the consultation and long term	[19]	11

^a^ The words ‘doctor’ and ‘physician’ were replaced by ‘health care provider’ thus translating the descriptions to health care in general

^b^ The segments of data of the articles are numbered in chronological order of publication year. Segments containing different themes are marked with a letter, e.g. 49a and 49b

## Footnotes

1 For reasons of readability ‘pharmacist’ was used instead of ‘health care provider’ in the description of the results.

2 The model was named the UMPA-model, where UMPA is an acronym of Utrecht’s Model for Patient centred Communication in the Pharmacy (in Dutch Apotheek). (UMPA also seems to be Gaelic for ‘about them’, which is an appropriate thought considering patient centred communication.)
